# Genomic Instability: The Driving Force behind Refractory/Relapsing Hodgkin’s Lymphoma

**DOI:** 10.3390/cancers5020714

**Published:** 2013-06-05

**Authors:** Hans Knecht, Christiaan Righolt, Sabine Mai

**Affiliations:** 1 Division d‘Hématologie, Département de Médecine, CHUS, Université de Sherbrooke, Québec, J1H 5N4, Canada; 2 Manitoba Institute of Cell Biology, The Genomic Centre for Cancer Research and Diagnosis, University of Manitoba, Winnipeg, Manitoba, R3E 0V9, Canada; 3 Department of Imaging Science and Technology, Delft University of Technology, 2628 CJ Delft, The Netherlands

**Keywords:** Hodgkin’s lymphoma, Reed-Sternberg cell, telomere, shelterin, 3D-FISH, nuclear architecture, genomic instability, nano-morphology

## Abstract

In classical Hodgkin’s lymphoma (HL) the malignant mononuclear Hodgkin (H) and multinuclear, diagnostic Reed-Sternberg (RS) cells are rare and generally make up <3% of the total cellular mass of the affected lymph nodes. During recent years, the introduction of laser micro-dissection techniques at the single cell level has substantially improved our understanding of the molecular pathogenesis of HL. Gene expression profiling, comparative genomic hybridization analysis, micro-RNA expression profiling and viral oncogene sequencing have deepened our knowledge of numerous facets of H- and RS-cell gene expression deregulation. The question remains whether disturbed signaling pathways and deregulated transcription factors are at the origin of refractory/relapsing Hodgkin’s lymphoma or whether these hallmarks are at least partially related to another major factor. We recently showed that the 3D nuclear organization of telomeres and chromosomes marked the transition from H- to RS-cells in HL cell lines. This transition is associated with progression of telomere dysfunction, shelterin disruption and progression of complex chromosomal rearrangements. We reported analogous findings in refractory/relapsing HL and identified the shelterin proteins TRF1, TRF2 and POT1 as targets of the LMP1 oncogene in post-germinal center B-cells. Here we summarize our findings, including data not previously published, and propose a model in which progressive disruption of nuclear integrity, a form of genomic instability, is the key-player in refractory/relapsing HL. Therapeutic approaches should take these findings into account.

## 1. Introduction

**Figure 1:**
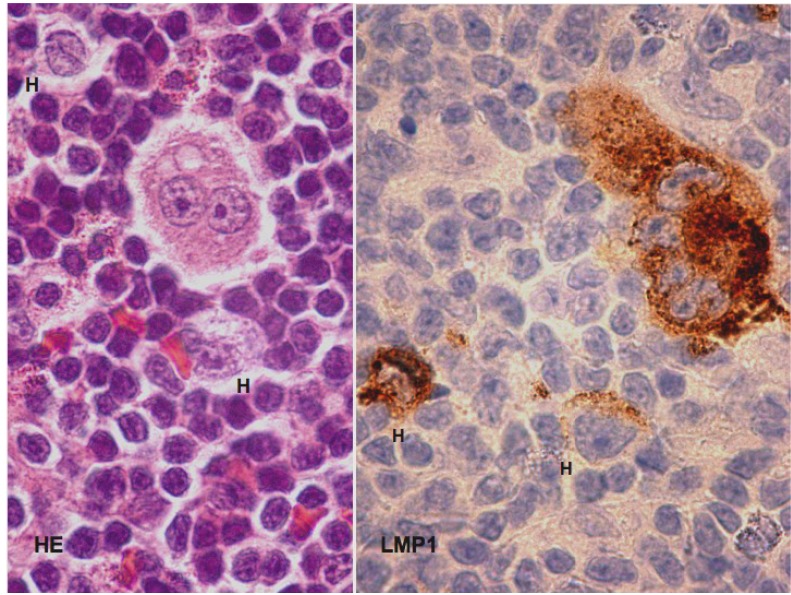
Mononuclear H-cells and bi/multinuclear diagnostic RS-cells. Hematoxilin-Eosin (HE) staining reveals two H-cells and one binuclear RS-cell (left). Immunostaining with anti-LMP1 antibody identifies two H-cells and one multinuclear RS-cell (right). In Western countries about 40% of HL cases express the EBV-encoded oncoprotein LMP1.

In the end of 2012 two detailed reviews including gene expression and functional data dealing with the molecular pathogenesis of HL have been published by members of the German Hodgkin Lymphoma Study Group [1,2]. The functional relevance of the microenvironment on disease progression has been reviewed by a Canadian group [3] and the impact of EBV [4] Notch and NF-κB signaling [5] on the pathogenesis of HL has also been described recently. In these reviews the tumour cells are identified as Hodgkin-Reed-Sternberg cells (HRS-cells), grouping mononuclear H-cells and bi/multinuclear RS-cells together. When assessing the nuclear architecture, it appears, however, fundamental to us to separate mononuclear H- from bi/multinuclear RS cells (Figure 1) since multi-nuclearity is a morphologic sign of disturbed nuclear integrity in HL [6,7].

## 2. Nuclear Architecture and Function

### 2.1. Chromosome Territories

The 3D nuclear architecture is closely related to cellular functions and chromosomes are organized in distinct territories [8,9]. Formal proof of these chromosome territories came from the identification of regular and inverted chromosomal architecture in rod cells associated with diurnal and nocturnal vision, respectively, in mammals [10].

2.2. 3D Organization of Telomeres

**Figure 2:**
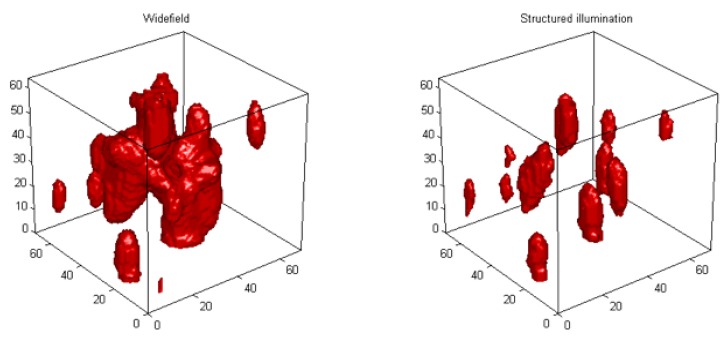
3D telomere analysis in the Hodgkin cell line HDLM-2. 3D superresolution imaging (3D-SIM) at a resolution up to 50nm dissects large aggregates into clusters of “t-stumps” and few small aggregates. Conventional microscopy (left) identifies one large central aggregate and three telomeres. 3D-SIM (right) dissects the central large aggregate into six telomeres and one smaller aggregate.

Telomeres are nucleoprotein complexes at the ends of chromosomes. Telomere DNA consists of multiple TTAGGG repeats ending in single stranded-overhang of the G-rich 3' strand [11]. Telomere organization is cell-cycle dependent and telomeres assemble into a disk in the G2 phase. In tumour cells this organization is, however, disturbed and telomere aggregates are formed [12]. We have developed quantitative software that enables us to measure the 3D nuclear organization of telomeres [13]. This allows us to determine the 3D nuclear localization and distribution of telomeres, their size, their numbers and the presence of telomere aggregates for each cell. Telomere aggregates are defined as clusters of telomeres that are found in close association and cannot be further resolved as separate entities at an optical resolution of 200 nm. They are hallmarks of cancer cells [14] and can be induced experimentally by c-Myc overexpression resulting in end-to-end telomere fusions of chromosomes at the origin of subsequent breakage-bridge-fusion (BBF) cycles resulting in genomic instability [15]. This genomic instability was also observed after *ex-vivo* EBV infection of human B-lymphocytes [16]. 3D superresolution imaging (3D-SIM) [17] identifies these aggregates partially as clusters of (extremely) small telomeres, so called “t-stumps” [18] a further marker of cancer cells (Figure 2).

2.2.1. 3D Organization of Telomeres in Hodgkin’s Lymphoma Cell Lines

In the HL-cell lines HDLM-2, L-428, L-1236, U-HO1 and U-H-HO1-PTPN1 mononuclear H-cells differ significantly from bi/multinuclear RS-cells concerning their nuclear volume (*p* < 0.0001) and the mass of telomeres/1,000 µm^3^ of nuclear volume (*p* < 0.01; except in U-HO-1) indicating a loss of telomere TAAGGG repeats during the transition from H-cells to RS-cells within each individual HL-cell line [19,20]. The number of telomere aggregates also increases (*p* < 0.03; except in L-428). The percentage of short and very short telomeres significantly progresses during the transition of H-cells to RS-cells in HDLM-2 (*p* < 0.0001) and L-1236 (*p* < 0.02) but is similar in the other HL-cell lines. It should be kept in mind that every HL-cell line has its own characteristics: for example, H- and RS-cells of U-HO1 have a total nuclear volume of 4,121 ± 1,350 µm^3^ and 15,301 ± 4,641 µm^3^, respectively (*p* < 0.0001), but H- and RS-cells of L-1236 have a total nuclear volume of 2,043 ± 728 µm^3^ and 5,335 ± 2,997 µm^3^, respectively (*p* < 0.0001). Thus the L-1236 HL-cell line has much smaller nuclei and the nuclear volume of an L-1236 RS-cell is not so much different from that one of a U-HO1 H-cell. Both cell lines do, however, have similar characteristics. Overall, the transition from H-to RS-cells is associated with profound changes in the 3D telomere dynamics. RS-cells show significantly fewer and shorter telomeres in relation to the total nuclear volume when compared to H-cells. In RS-cells telomere-poor “ghost” nuclei are often adjacent to nuclei with huge telomere aggregates indicating a major problem in chromosomal separation during mitosis [19]. 

We extended our initial studies to the nuclear chromosome organization in H-cells and RS-cells using interphase chromosome painting, spectral karyotyping (SKY), and 3D-SIM [21]. Interphase chromosome painting confirmed the generation of “ghost” nuclei and giant “zebra” chromosomes in RS-cells generated by BBF-cycles. SKY documented the increasing complexity of chromosomal rearrangements during the progression from H-cells to RS-cells leading to chromosomes including up to seven different chromosomal partners. 3D-SIM revealed intra-nuclear chromosome bridges between nuclei of RS cells [21]. These findings convincingly show that genomic instability is a driving force in the generation of H-cells and RS-cells. It should be kept in mind that the HL-cell lines are derived from primary refractory or relapsing HL and thus reflect the settings of aggressive HL *ex vivo*.

2.2.2. 3D Organization of Telomeres in Hodgkin’s Lymphoma Biopsies

**Figure 3:**
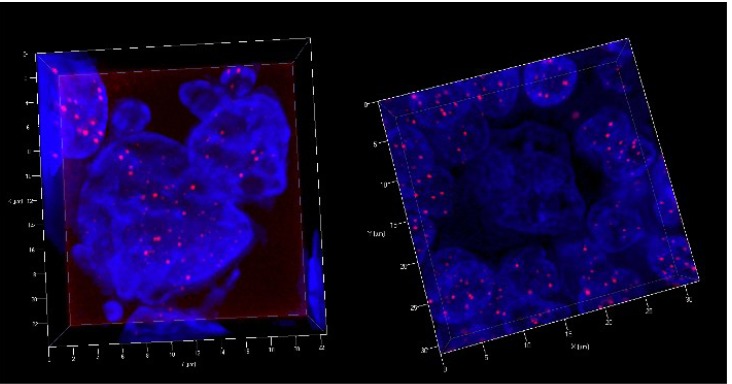
Combined 3D nuclear staining (telomeres red; nuclear DNA blue) shows a bi-nucleated RS-cell (left) with three small satellite nuclei and high telomere number. A typical “ghost” RS-cell surrounded by a corona of lymphocytes is shown at the right. In both cases the patients entered rapid, still ongoing, remission.

The results obtained from lymph node biopsies of HL patients correlate largely with those of the HL-cell lines and there is no difference to LMP1 positive HL cases [19,22]. RS-cells with high telomere mass as well as RS-cells with “ghost nuclei” are regularly observed and thus confirm ongoing nuclear architecture remodeling (Figure 3).

## 3. 3D Telomere Dynamics in Translational Oncology

**Figure 4:**
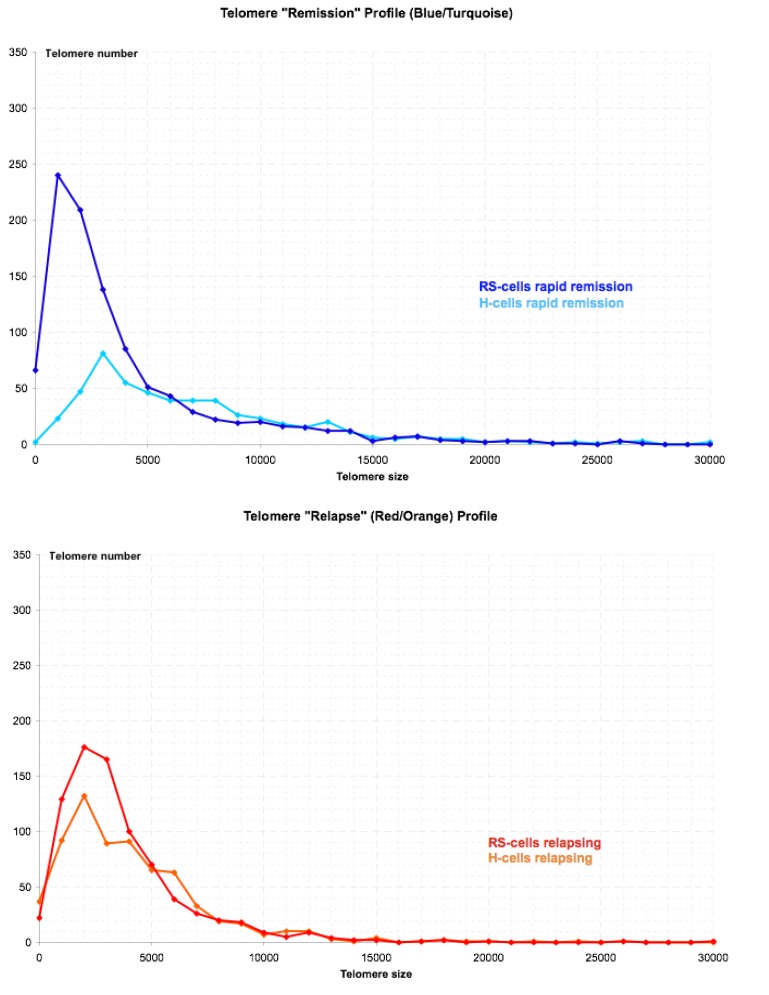
Typical telomere profiles at first diagnostic biopsy associated with rapid remission (top) and relapsing/refractory disease (bottom). Telomere distribution according to size in mononuclear H-cells and bi/multinuclear RS-cells. Results are based on 3D analysis of 30 Hodgkin and 30 Reed-Sternberg cells in a 5 μm lymph node section of the diagnostic biopsy. Frequency (y-axis) and relative fluorescent intensity, *i.e.*, size of telomeres (x-axis), are plotted against each other.

We previously performed 3D telomere FISH on 50 patients with HL in a clinical study. Two specific 3D telomere FISH patterns (established on 3D nuclear analysis of 30 H-cells and 30 RS-cells in each case) emerged at diagnosis [23]. The first pattern is associated with rapid and long lasting remission and is characterized by a rather low number (<40%) of very small telomeres (up to 5,000 U) in the mononuclear H-cells, the RS-cells generally displaying a high number (>60%) of very small telomeres. The second pattern is associated with progression/relapse and characterized by a high number of very small telomeres already in the mononuclear H-cells (>60%) who have a similar telomere profile to the multinucleated RS-cells. This is consistent with fact that these mononuclear H-cells have already undergone multiple rounds of replication including multiple BBF-cycles but still retain the capacity of complete cellular division in spite of accumulated chromosomal changes. H-cells with a low number of very small telomeres appear, however, to replicate less rapidly and are far away from end-stage RS cells with complex chromosomal rearrangements. A typical “remission¨ and a typical “relapse” profile are shown in Figure 4.

**Figure 5:**
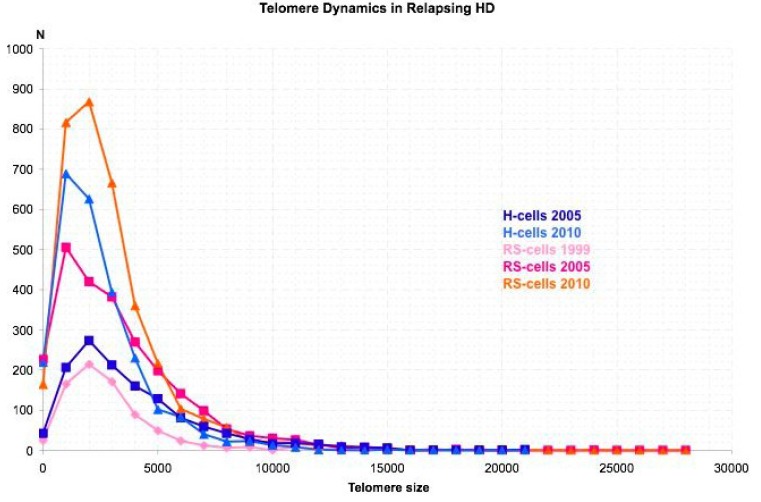
Typical telomere profiles associated with relapsing and finally refractory disease.Telomere distribution according to size in mononuclear H-cells and bi/multinuclear RS-cells. Results are based on 3D analysis of 30 Hodgkin and 30 Reed-Sternberg cells in a 5μm lymph node section of each diagnostic biopsy. Frequency (y-axis) and relative fluorescent intensity, *i.e.*, size of telomeres (x-axis), are plotted against each other. As a particularity, the initial diagnostic biopsy had numerous RS-cells but only few H-cells and the number of aggregates was low but increased fivefold in the last biopsy.

In relapsing and finally refractory HL two evolutions of telomere dynamics are observed. The most frequent shows an increasing number of aggregates and very short telomeres with every relapse, consistent with ongoing BBF-cycles (Figure 5). Low telomere numbers consistent with pronounced hypoploidy were observed in a patient with rapid pulmonary relapse after autologous bone marrow transplantation and radiation therapy.

We compared 10 diagnostic biopsies (prior to any treatment) of 10 patients entering rapid and long lasting remissions (median 48 months) with 10 diagnostic biopsies (prior to any treatment) of 10 patients with progressing/relapsing disease. All but 3 patients (two children, one adult with preexisting pulmonary disease) had identical first line ABVD chemotherapy. When compared to H-cells of patients entering rapid remission, H-cells of patients with progressing/relapsing disease showed significantly more very small telomere (65.6 ± 10.2% *versus* 39.9 ± 15.6%, *p* < 0.01) and aggregates (2.50 ± 0.87 *versus* 1.85 ± 1.16, *p* < 0.02) as well as a decrease of mean telomere intensity (definitions of these parameters according to [23]). Detailed clinical information about these two groups of patients is given in Table 1, Table 2. “Rapid” (Table 1) denotes the number of chemotherapy cycles applied until metabolic remission was confirmed by fluorodeoxyglucose (FDG) positron emission tomography-computed tomography (PET-CT) and “1st Line” (Table 2) denotes the number of chemotherapy cycles applied until a first metabolic remission or disease progression was identified by PET-CT.

**Table 1:** Clinical data and percentage of very small telomeres of mononuclear Hodgkin cells at diagnosis (mean 39.9%) in patients with rapid and long ongoing remission (mean 57 months).

Case	Sex	Age	Type	LMP1	Stage	Rapid	0–5,000 u
							very small telomeres (%)
1	F	57	MC	Pos	IIIA	4 ABVD	39.3
2	M	24	MC	Pos	IIIB	1 ABVD	37.3
3	M	42	MC	Neg	IIA	2 ABVD	27.8
4	M	19	SN	Neg	IVA	2 ABVD	35.9
5	F	28	SN	Pos	IIA	4 ABVD	32.7
6	M	27	SN	Neg	IIIs	4 ABVD	46.4
7	M	34	SN	Neg	IIIB	4 ABVD	16.7
12	M	71	MC	Pos	IIIB	4 ABVD	34.3
16	F	73	NS	Neg	IIIB	4 ABVD	78.6
11	M	17	NS	Neg	IIB bulky	4 DBVE-PC+IFRT	49.8

**Table 2:** Clinical data and percentage of very small telomeres of mononuclear Hodgkin cells at diagnosis (mean 65.6%) in patients with progressive or relapsing disease.

Case	Sex	Age	Type	LMP1	Stage	1st Line	0–5,000 u
							very small telomeres (%)
17a	F	77	NS	Pos	IIIB	4 AVBD	62.3 progressive
19a	M	61	NS	Neg	IIIB	6 ABVD	68.2 early relapse
20a	M	38	NS	Neg	IVB	6 ABVD	77.9 late relapse
21a	M	21	NS	Neg	IIIB	4 AVBD	48.1 progressive
22a	M	49	NS	Neg	IIIB	6 AVBD	71.4 late relapse
23a	F	14	NS	Neg	IVA	4 DVBE-PC + IFRT	77.6 early relapse
25a	M	48	NS	Neg	IVB	6 ABVD	52.9 early relapse
26a	M	28	NS	Neg	IIB	4 ABVD	54.0 early relapse
10a	F	35	NS	Neg	IIIsB	2 CVPP-A0	74.1 late relapse
40a	F	44	NS	Pos	IVB	2 ABVD	69.9 progressive

We hypothesize that for such aggressive HL the 3D telomere FISH profile indicates advanced genomic instability of still mononuclear H-cells and points to their very dangerous behaviour. 

## 4. EBV Is probably Not an Innocent Bystander

The EBV encoded LMP1 oncogene targets the shelterin-complex through transcriptional and translational repression of TRF1, TRF2 and POT1 in a reversible manner [24]. However, the molecular mechanisms of this reversible repression are still under investigation. *Ex vivo* EBV infection of human lymphocytes induces telomere dysfunction through telomere end fusions and telomere-free chromosome ends [16]. EBNA1 promotes telomere dysfunction via induction of oxidative stress [25], and through tethering of TRF1 associated tankyrase [26]. Moreover, H-cells and RS-cells harbour LMP1 variants with higher oncogenic potential [27] and very high NF-κB activation potential [28]. There is accumulating evidence that in EBV-associated HL this virus acts as a double cutting sword. First, mitotic activity is boosted through NF-κB activation. And second, genomic instability appears to be generated through targeting the telomere-shelterin complex. 

## 5. The Telomere-Shelterin Complex: Achilles Tendon of Relapsing/Refractory HL

In both, HL-cell lines and patient lymph node biopsies we identified shelterin dysfunction, centrosome amplifications and multipolar spindle formation in dividing RS-cells, which indicates disturbed 3D nuclear organization [19]. Support for our hypothesis comes from a recent single cell based gene expression study on HRS-cells [29], who identified a massive down-regulation of STAG3, a component of the cohesin complex needed for chromosomal stability, and a tremendous up-regulation of TUBB2A and TUBB2B, two tubulin related genes. A further gene implicated in mitosis, KLHDC8B, encoding a Kelch protein, is also disturbed in HL [30]. Further support for our hypothesis of the telomere-shelterin complex as the “plaque tournante” in the pathogenesis of HL comes from a most interesting recent case report [31]: Lymphocyte predominant nodular Hodgkin’s lymphoma (LPNHL) and classical HL co-occurred in the same lymph nodes of a patient. Both, tumour cells of LPNHL and classical HL had identical variable, diversity and joining (VDJ) immunoglobulin heavy chain rearrangements and somatic hypermutation events. This demonstrates the same clonal origin. EBV genomes with the expression of the LMP1 oncoprotein were, however, only identified in the RS-cells of classical HL but not in the tumour cells of LPNHL. This is best explained by EBV infection of a part of the common precursor cells with subsequent attack of EBV encoded proteins on the telomere-shelterin complex leading to development of HL with diagnostic RS-cells through induction of genomic instability.

## 6. Conclusions

Recent results from our group indicate a new direction in the molecular pathogenesis of HL. Apart from the well-established up-regulated NF-κB, Notch1 and JAK/STAT signaling pathways a common molecular pathogenetic denominator emerges: The telomere shelterin-complex and the spindle apparatus. Vulnerability of the telomere-shelterin complex, either targeted by EBV infection, c-myc activation or still unknown factors, induce profound chromosomal changes through initiation of repeated mitotic BBF-cycles. This results in progressive 3D nuclear disorganisation as heralded by various forms of mononuclear H-cells and multinucleated RS-cells with remodeled “composite” chromosomes. Advanced genomic instability is therefore a hallmark of refractory/relapsing HL. New therapeutic approaches targeting these components are successful in clinical settings that had proven unfavourable until recently [32]. 
